# Harm reduction services and interventions for People Who Use Drugs (PWUD) in Latin America and the Caribbean (LAC) between 2013–2024: A scoping review protocol

**DOI:** 10.1371/journal.pone.0334978

**Published:** 2025-11-24

**Authors:** Ignacio Bórquez, Katie Bailey, Gregory Laynor, Lidiane Toledo, Francisco I. Bastos, Julian Santaella-Tenorio, Álvaro Castillo-Carniglia, Magdalena Cerdá, Noa Krawczyk

**Affiliations:** 1 Center for Opioid Epidemiology and Policy (COEP), Department of Population Health, Division of Epidemiology, NYU Grossman School of Medicine, New York, New York, United States of America; 2 University of California San Diego, San Diego, California, United States of America; 3 NYU Health Sciences Library, NYU Grossman School of Medicine, New York, NY, United States of America; 4 Fundação Owaldo Cruz (FIOCRUZ), Rio de Janeiro, Brazil; 5 Departamento de Salud Pública y Epidemiología, Pontificia Universidad Javeriana, Calí, Colombia; 6 Departamento Nacional de Salud Pública, Facultad de Medicina y Ciencia, Universidad San Sebastián, Santiago, Chile; Public Library of Science, UNITED KINGDOM OF GREAT BRITAIN AND NORTHERN IRELAND

## Abstract

**Introduction:**

In Latin America and the Caribbean (LAC) the response to substance use has primarily been abstinence-based, acute-care-oriented treatments. While harm reduction services (HRS) and interventions have expanded in LAC over the last decade, the research evidence on such programs has been sparse and disjointed.

**Objective:**

This scoping review will map peer-reviewed literature on HRS and interventions in LAC, and synthesize gaps and opportunities for policy, practice, and research.

**Inclusion criteria:**

Studies conducted in LAC. The HRS that will be included in the search are opioid agonist therapy, syringe services programs, drug consumption facilities, safer consumption kits, managed alcohol programs, and drug-checking services. The scoping review will consider peer-reviewed original research, including qualitative, quantitative, and mixed-methods designs. We will exclude studies addressing harms associated with nicotine or tobacco use. We included original research written in English, Spanish, Portuguese, or French published between January 2013 and December 2024.

**Methods:**

We will conduct literature searches in English (PubMed, Scopus, Web of Science), Spanish, Portuguese (SciELO and BIREME), and French (BIREME). Two reviewers will independently screen the literature. Extraction of characteristics of the studies using a template in Covidence. Data on the HRS and interventions studied and implemented in LAC will be summarized and presented in tables, graphs, and a narrative summary. We will use a narrative synthesis approach to summarize implications for policy, research, and practice identified in the literature. The review was registered in Open Science Framework (https://osf.io/qya7c/),

**Discussion:**

The proposed scoping review will provide valuable information regarding the current state of HRS and interventions for PWUD in LAC. This in return can help guide future research for evaluating services that are already being implemented or unveil services needed in the region. To our knowledge, this is the first scoping review to map HRS in LAC using a systematic approach. Furthermore, among the strengths of this review are: the broad number of services, countries, and time, as well as the consultation with experts and knowledge users.

## 1. Introduction

Alcohol and drug use pose an important burden on population health and communities around the globe. In 2016, an estimated 99.2 million disability-adjusted life years (DALYs) were attributable to alcohol use, whereas 31.8 were to drug use as a risk factor, accounting for approximately 5.5% of all DALYs [1]. These burdens vary greatly across the globe. The Latin American and Caribbean region (LAC) includes 33 countries and 15 territories [2]. A common history of colonization and neocolonialism, alongside a wide range of ethnic, cultural, and geographic diversity, has resulted in some shared, yet distinct drug use patterns [3,4], governmental policies [5], and civil society responses regarding substance use and substance use disorders (SUDs).

Most cocaine is produced in the Andes mountain range in South America [6], and its derivatives -like crack cocaine or cocaine paste- are particularly burdensome in the region [3]. Alcohol is also responsible for a significant burden, with LAC having one of the highest regional numbers of years lost due to alcohol use disorder globally [7]. Alcohol and cocaine polysubstance use is common because of its synergic effects [8] and is exceptionally damaging [9,10]. Recent data shows an increase in overdose related to psychotropic medication, leading to a rise in hospitalization in countries like Colombia [11]. Moreover, a “new” psychoactive drug called “Tusi” or “pink cocaine” has emerged in LAC countries. Tusi is usually a mix of various substances, including ketamine, MDMA, GHB, cocaine, and caffeine, an attempt to imitate the synthetic 2-CB party drug which is used primarily in Europe [12]. Rates of opioid and injection drug use (IDU) are relatively low [3,4,13], however, LAC has one of the highest regional prevalences of HIV among people who inject drugs, with 37.5% versus 17.8% globally [13]. Furthermore, there is a new and growing trend of opioid use in countries like Colombia [14] and on the border between Mexico and the United States [15].

The policy response to substance use in LAC has been heavily influenced by the United States’ “War on Drugs” model [16], which criminalizes drug use and has led to the overrepresentation of people who use drugs (PWUD) in the criminal legal system [17]. Most LAC health systems primarily provide acute-care-oriented treatments to people with SUD through public and private institutions [18], particularly in the form of therapeutic communities that generally follow an abstinence-based discourse around SUDs [19,20]. These communities are often independent of government oversight and maintain autonomy in their clinical practices [21]. A few countries in LAC (i.e., Brazil) stand out by offering psychosocial care networks to support PWUD within the healthcare system, though these networks coexist with therapeutic communities [22].

Harm reduction is an alternative approach to substance use and commonly refers to policies, programs, and practices that aim to minimize the negative consequences of substance use and related criminalizing laws as opposed to requiring abstinence [23,24]. When applied to healthcare, it involves services and interventions that are provided using a non-judgmental approach, where people can receive support without discrimination, coercion, or abstinence from substance use as a requisite [23]. It is also a pragmatic approach: it uses a package of evidence-based interventions and policies to reduce the harmful effects of substance use, which are tailored for particular populations and contexts [25]. Examples of harm reduction services (HRS) include, but are not limited to, opioid agonist therapy (OAT), syringe services programs (SSPs), drug consumption facilities [25], provision of safe-use kits [26,27], and managed alcohol programs [28]. Moreover, they are typically community-based and/or peer-led and offer additional goods and services like food, hygiene utilities, and clothing, aiming to promote community, health, and referral to other services for PWUD [25].

As HRS grew globally, largely as a public health response due to the HIV/AIDS epidemic associated with injection drug use (IDU) [29,30], their implementation has been relatively rare in LAC [31,32] and for non-IDU purposes [26,28,33,34]. Instead, LAC harm reduction is mostly implemented as self-regulating practices within the “formal” treatment system, and community and peer-led organizations are exceptional, although they have burgeoned in the past decade [31,35]. As scarce as HRS are in LAC, the scientific evidence on these services is even more sparse. Most studies on harm reduction strategies, services, interventions, and models originate from the Global North. A deeper understanding of the specificities and challenges of harm reduction in LAC could assist managers and policymakers in developing strategies and guidelines tailored to the region’s unique needs. This scoping review aims to map peer-reviewed literature on HRS and interventions in LAC for PWUD between 2013 and 2024 and synthesize the main gaps in policy, practice, and research.

## 1. Materials and methods

Scoping reviews are a systematic way of mapping evidence on a topic to identify key concepts, theories, sources, and knowledge gaps [36]. They can be conducted for various objectives, such as examining the size, variety, and characteristics of evidence on a topic, determining the value of conducting a systematic review, summarizing findings from a diverse range of knowledge, or identifying gaps in the literature to aid future research planning [36]. This scoping review will be conducted according to the framework developed by Arksey and O’Malley, which consists of: 1) identifying the research questions, 2) identifying relevant studies, 3) study selection, 4) charting and collecting the data, 5) summarizing and reporting results, and 6) conducting consultations [37]. The results will be reported following the Preferred Reporting Items of Systematic Reviews and Meta-Analyses for Scoping Review (PRISMA-ScR) checklist [38] and it is registered on Open Science Framework (https://osf.io/qya7c/). The adapted PIRSMA-P checklist for review protocols is available in the Appendix. All amendments to the protocol will be registered, declared, and explained in the final manuscript.

### Stage 1: Identifying the research question

Our main research questions were formulated as follows:

1. What HRS and interventions for PWUD have been studied and implemented in LAC?
2. What is the geographical distribution of these services and interventions in LAC?
3. Who are their main implementers? What are the characteristics of the implementers (such as SUDs treatment staff, researchers, and community-based organizations, among others)?
4. Who has funded such services (e.g., local or national governments, private donations)?
5. What are the main policy, practice, and research gaps around HRS and interventions in LAC?

### Stage 2: Identifying relevant studies (eligibility criteria)

To formulate a comprehensive review of HRS and interventions in LAC, the search strategies will cover original research studies in peer-reviewed literature in indexed databases. Although there is a variety of definitions and possible HRS and interventions, we will focus on substance-use-related services: (1) OAT; (2) SSPs; (3) drug consumption facilities; (4) safer consumption kits; (5) managed alcohol programs; and (6) drug checking services. We will include only services and interventions that explicitly endorse “harm reduction” or “non-abstinence” approaches to care of PWUD. We will exclude harm reduction efforts for tobacco use as they usually rely on products that are available in the market, such as e-cigarettes or smokeless tobacco (e.g. patches, gum, etc.) [39], as opposed to HRS for alcohol and other substances. S1 Table in the Appendix lists the countries and overseas territories to be included. We will incorporate records published from January 2013 to December 2024 to identify recent efforts. We will exclude sources not written in English, Spanish, Portuguese, or French. Inclusion and exclusion criteria are presented in Table 1.

**Table 1:** Inclusion and exclusion criteria.

Population	Concept	Context	Type of Sources
The target population includes individuals who use drugs or alcohol in Latin America and the Caribbean.	Studies about health services or interventions for substance use with an explicit harm reduction approach or that don’t require abstinence to receive support. This includes but is not limited to (1) opioid agonist therapy; (2) syringe services programs; (3) drug consumption facilities; (4) safer consumption supplies or kits; (5) managed alcohol programs; and (6) drug checking and testing services.	Healthcare facilities (hospitals, primary care clinics, substance use treatment), community-based organizations.	Peer-reviewed literature, including quantitative, qualitative, or mixed- method designs. We will exclude sources not written in English, Spanish, Portuguese, or French. We will include sources published between January 2013 and December 2024.

We developed our search strategies by selecting specific keywords based on the countries and overseas territories, population, and HRS. These keywords will be validated according to the Medical Subject Headings (MeSH) of the U.S. National Library of Medicine (NLM) and the Health Sciences Descriptors (DeCS) at the Latin American and Caribbean Center on Health Sciences Information (BIREME), which is derived from MeSH. This will enable us to include relevant keywords in Portuguese, Spanish, and French languages.

We will conduct a thorough search for peer-reviewed papers in established databases, including PubMed, Scopus, and Web of Science Core Collection (1900-present). We will search in repositories of indexed scientific work in the LAC region: the Scientific Electronic Library Online (SciELO) and the BIREME. The collected documents will be organized in a specific table according to the database. Bibliographic free software will be used to organize the references (Zotero). With the final list of peer-reviewed articles, we will further review their references (backward chaining) and citations (forward chaining) using the Citationchaser software [40]. Table 2 provides the search strategy using keywords and queries in English for Pubmed, for other databases please review the S2 Table in the Appendix. Queries in Spanish, Portuguese, and French are shorter due to the limitations of one of the used databases, however, they cover all territories, HRS, and populations.

**Table 2:** Keywords and queries for search strategy of harm reduction services in LAC for PubMed.

Database	Keywords and Queries
PubMed	((“Latin America”[tiab] OR “South America”[tiab] OR “Central America”[tiab] OR “Caribbean”[tiab] OR “Argentina”[tiab] OR “Belize”[tiab] OR “Bolivia”[tiab] OR “Brazil”[tiab] OR “Chile”[tiab] OR “Colombia”[tiab] OR “Costa Rica”[tiab] OR “Ecuador”[tiab] OR “El Salvador”[tiab] OR “Guatemala”[tiab] OR “Guyana”[tiab] OR “Honduras”[tiab] OR “Mexico”[tiab] OR “Nicaragua”[tiab] OR “Panama”[tiab] OR “Paraguay”[tiab] OR “Peru”[tiab] OR “Suriname”[tiab] OR “Uruguay”[tiab] OR “Venezuela”[tiab] OR “Antigua and Barbuda”[tiab] OR “The Bahamas”[tiab] OR “Barbados”[tiab] OR “Cuba”[tiab] OR “Dominica”[tiab] OR “Dominican Republic”[tiab] OR “Grenada”[tiab] OR “Haiti”[tiab] OR “Jamaica”[tiab] OR “Saint Kitts and Nevis”[tiab] OR “Saint Lucia”[tiab] OR “Saint Vincent and the Grenadines”[tiab] OR “Trinidad and Tobago”[tiab] OR “Anguilla”[tiab] OR “Aruba”[tiab] OR “Bermuda”[tiab] OR “British Virgin Islands”[tiab] OR “Caribbean Netherlands”[tiab] OR “Cayman Islands”[tiab] OR “Curacçao”[tiab] OR “Falkland Islands”[tiab] OR “French Guiana”[tiab] OR “Guadeloupe”[tiab] OR “Martinique”[tiab] OR “Montserrat”[tiab] OR “Puerto Rico”[tiab] OR “Saint Barthélemy”[tiab] OR “Saint Martin”[tiab] OR “South Georgia and the South Sandwich Islands”[tiab] OR “Turks and Caicos”[tiab] OR “U.S. Virgin Islands”[tiab] OR “Latin America”[Mesh] OR “Caribbean Region”[Mesh] OR “Central America”[Mesh] OR “South America”[Mesh])) AND ((“People who use drug*”[tiab] OR “People who inject drug*”[tiab] OR “People who use drug*”[tiab] OR “PWID”[tiab] OR “PWUD”[tiab] OR “PWIDs”[tiab] OR “PWUDs”[tiab] OR “Substance Abuse”[tiab] OR “Substance Dependence”[tiab] OR “Substance Use Disorder*”[tiab] OR “Substance use”[tiab] OR “Alcohol*”[tiab] OR “Cannabi*”[tiab] OR “Marijuana*”[tiab] OR “Cocaine*”[tiab] OR “Freebase”[tiab] OR “Coca base”[tiab] OR “Coca”[tiab] OR “Coca paste”[tiab] OR “Crack cocaine”[tiab] OR “Amphetamine*”[tiab] OR “Methamphetamine*”[tiab] OR “MDMA”[tiab] OR “Ecstasy”[tiab] OR “Psychoactive substance*”[tiab] OR “Hallucinogen*”[tiab] OR “Ketamine*”[tiab] OR “Sedatives”[tiab] OR “Benzodiazepine*”[tiab] OR “Psilocybin*”[tiab] OR “LSD”[tiab] OR “Opioid*”[tiab] OR “AUD”[tiab] OR “OUD”[tiab] OR “SUD”[tiab] OR “Alcohol-Induced Disorders”[Mesh] OR “Alcohol-Related Disorders”[Mesh] OR “Amphetamine-Related Disorders”[Mesh] OR “Cocaine-Related Disorders”[Mesh] OR “Drug Users”[Mesh] OR “Inhalant Abuse”[Mesh] OR “Marijuana Abuse”[Mesh] OR “Opioid-Related Disorders”[Mesh] OR “Substance Withdrawal Syndrome”[Mesh] OR “Substance-Related Disorders”[Mesh])) AND ((“Harm reduction”[tiab] OR “Risk reduction”[tiab] OR “Harm-reduction”[tiab] OR “Risk-reduction”[tiab] OR “Buprenorphine”[tiab] OR “Medication Assisted Treatment”[tiab] OR “Methadone”[tiab] OR “MMT”[tiab] OR “Naltrexone”[tiab] OR “Opiate Medication-Assisted Therap*”[tiab] OR “Opiate Medication-Assisted Treatment*”[tiab] OR “Opiate Replacement Therap*”[tiab] OR “Opiate Replacement Treatment*”[tiab] OR “Opiate Substitution Therap*”[tiab] OR “Opiate Substitution Treatment*”[tiab] OR “Opioid Medication-Assisted Therap*”[tiab] OR “Opioid Medication-Assisted Treatment*”[tiab] OR “Opioid Replacement Therap*”[tiab] OR “Opioid Replacement Treatment*”[tiab] OR “Opioid Substitution Therap*”[tiab] OR “Opioid Substitution Treatment*”[tiab] OR “OST”[tiab] OR “Needle and syringe program*”[tiab] OR “Needle Exchange*”[tiab] OR “Needle-Exchange*”[tiab] OR “NEP”[tiab] OR “SEP”[tiab] OR “SSP”[tiab] OR “Syringe Exchange*”[tiab] OR “Syringe Services*”[tiab] OR “Syringe-Exchange*”[tiab] OR “Drug consumption facilit*”[tiab] OR “Drug consumption room*”[tiab] OR “Drug consumption site*”[tiab] OR “Overdose prevention center*”[tiab] OR “Supervised consumption*”[tiab] OR “Supervised Injecting*”[tiab] OR “Drug Paraphernalia”[tiab] OR “Foil*”[tiab] OR “Mouthpiece*”[tiab] OR “Pipe*”[tiab] OR “Safer consumption equipment*”[tiab] OR “Safer consumption kit*”[tiab] OR “Safer consumption suppl*”[tiab] OR “Safer crack”[tiab] OR “Safer drug use”[tiab] OR “Safer smoking equipment*”[tiab] OR “Safer smoking kit*”[tiab] OR “Safer use equipment*”[tiab] OR “Safer use kit*”[tiab] OR “Safer use suppl*”[tiab] OR “Sterile consumption equipment*”[tiab] OR “Sterile consumption kit*”[tiab] OR “Sterile consumption suppl*”[tiab] OR “Sterile smoking equipment*”[tiab] OR “Sterile smoking kit*”[tiab] OR “Sterile use equipment*”[tiab] OR “Sterile use kit*”[tiab] OR “Sterile use suppl*”[tiab] OR “Alcohol management program*”[tiab] OR “Alcohol Moderation Management”[tiab] OR “Moderation Management”[tiab] OR “Managed alcohol program*”[tiab] OR “Managed alcohol”[tiab] OR “Alcohol Moderation”[tiab] OR “Community managed alcohol program”[tiab] OR “Drinking under control program*”[tiab] OR “Drinkers lounge”[tiab] OR “Managed alcohol administration”[tiab] OR “Drug checking*”[tiab] OR “Drug safety test*”[tiab] OR “Drug test*”[tiab] OR “Reagents*”[tiab] OR “Street drug analys*”[tiab] OR “Test strip*”[tiab] OR “Testing of illicit substance*”[tiab] OR “Testing of substance*”[tiab] OR “Harm Reduction”[Mesh] OR “Needle-Exchange Programs”[Mesh] OR “Opiate Substitution Treatment”[Mesh] OR “Community Health Services”[Mesh]))

### Stage 3: Study selection

**Fig 1:**
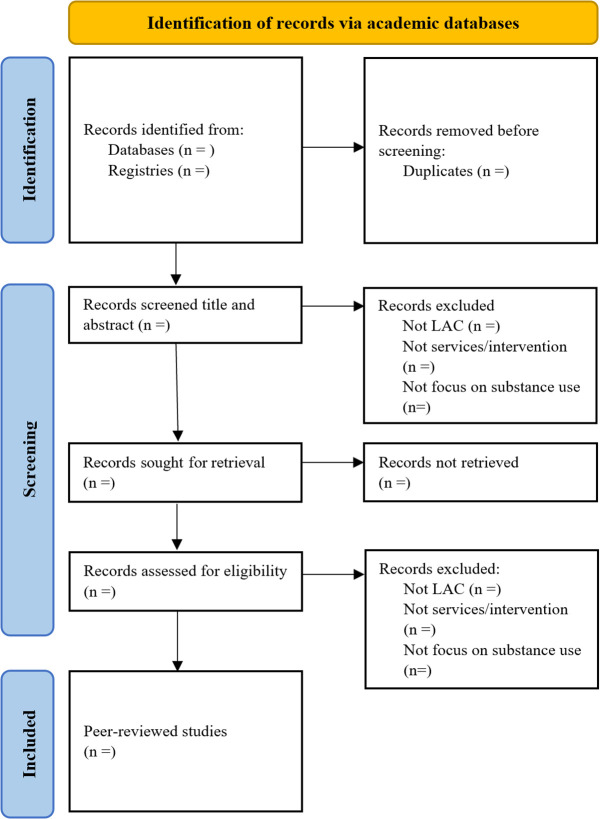
Flow chart for the study selection.

We will follow a two-step approach. Firstly, duplicate publications will be removed from consideration, as shown in Fig 1. Secondly, two authors will independently screen the titles and abstracts of the identified documents based on the selection criteria using Covidence. We will include services and interventions that explicitly endorse harm reduction, as well as those that do not use harm reduction terminology per se but suggest that they do not employ an abstinence-only approach to care and ultimately focus on reducing harms of substance use. The two reviewers will have experience with HRS in LAC and will take a conservative approach when screening. We will include studies where the potential for harm reduction is uncertain or not explicitly stated, considering that this information may not be disclosed due to political or funding concerns. We will exclude documents with data from countries outside of LAC, those that do not focus on HRS services or interventions for substance use (e.g., oriented to tackle the harms associated with the supply of substances or with traditional treatment programs), or articles describing protocols of studies with no results. If the two reviewers have different opinions on whether a study meets the eligibility criteria, a third reviewer will be involved to assess the study and facilitate a consensus decision.

### Stage 4: Extracting and charting data

Each selected document will undergo a full-text review by two reviewers. Then one reviewer will extract the information, chart the data independently, discuss the results, and update the data-charting process iteratively. Data extraction will be performed using the information included in Table 3. All text extractors will undergo a piloting of three random records to compare the used criteria while extracting. Because of the wide variety of services provided by harm reduction programs, we will also record if other social and health services not included in our search terms are being provided within the identified records. For instance, infectious disease testing and treatment, psychosocial support, safe sex interventions, and drop-in-center, among others.

**Table 3:** Extraction data table for peer-reviewed literature.

Field	Description
**ID**	Number of reference
**Start date of extraction**	MM/DD/YYYY
**End date of extraction**	MM/DD/YYYY
**Title**	Article’s title
**Year**	2013 to 2024
**Comments**	Free text
**First author’s last names**	First author’s last names. If two last names, include both
**Institutional affiliations of the first author**	Affiliations of the first author
**Country**	The country where the study data was recollected
**Involves multiple countries**	Yes/No/Unclear
**Other countries**	List of other countries considered
**Specific location (city or geographic area)**	A specific location within the country where data was recollected. This might be a city, region, or state.
**Aim**	Article’s main aim
**Study design**	Randomized control trial (RCT); Non-randomized experimental study; Cohort study; Cross-sectional study; Case-control study; Qualitative research (e.g., workshops, focus groups, ethnography); Mixed methods (Quantitative + Qualitative); Case series; Case report; Economic evaluation; Descriptive (no design, commentary on bibliography); Not stated; Other (free text)
**Study name**	Name of the study (if stated).
**Time period/duration of data recollection**	The period in which data was recollected (MM/YYYY-MM/YYYY).
**Primary setting in which service or intervention is delivered**	Community-based organizations (NGOs); SUD treatment centers; Hospitals; Primary care; Street-based outreach (e.g., streets, parks, shelters, motels, and vacant); Not stated; Other (free text)
**Implementers of the service or intervention**	Government; Private institutions; Healthcare workers; SUD treatment staff; Community-based organizations (NGOs); Researchers; Peers or people who use drugs (PWUD); Not stated; Other (free text)
**Target population description**	Description of the target population included in the study.
**Total sample size**	Sample size of the study (if applicable).
**Primary substance used by the study population/service looks to reduce harm.**	Opioids; Alcohol; Cocaine powder; Smokable cocaine (e.g., crack or free base); Amphetamines/Methamphetamines; Cannabis; Not clear; Other (free text)
**Other substances used by the study population**	Opioids; Alcohol; Cocaine powder; Smokable cocaine (e.g., crack or free base); Amphetamines/Methamphetamines; Cannabis; Not clear; Other (free text)
**Includes people who inject drugs (PWID)**	Yes/No/Unclear
**Services and/or interventions**	Opioid agonist therapy (e.g., methadone, naltrexone, buprenorphine); Other substitution therapy (e.g., for alcohol); Syringe service programs; Drug consumption facilities; Safer consumption supplies or kits; Managed alcohol programs; Drug checking and testing services; Other (free text)
**Description of the service or intervention**	Free text
**Related interventions described in the text**	Free text
**Main Outcomes**	Outcomes, intended and unintended effects
**Harm reduction definition**	If harm reduction is mentioned in the text, extract all the sentences in which researchers used it.
**Abstinence and non-abstinence**	If the words abstinence or non-abstinence are mentioned in the text, extract all the sentences in which researchers used it.
**HR Definition: implicit or explicit**	Implicit (does not define the concept but names it); Explicit (document explicitly defines harm reduction); Not stated.
**Other concepts**	Extract all sentences in which other concepts that the authors relate to harm reduction appear in the text.
**Primary findings as reported in the abstract**	Description of the primary findings of the study. If the study has no abstract, extract from the results section.
**Policy, practice, and research gaps**	Extract all sentences where authors describe gaps in policy, practice, and research.
**Funding sources of the study**	Free text
**Funding sources of the service**	Free text
**Related studies**	References to related studies in LAC are included in the introduction or discussion section of the articles.
**Other comments**	Any additional comments of the study (add when fully reviewed)

### Stage 5: Data analysis, synthesis, and discussion of results

To respond to the first four research questions (which aim to describe the HRS and interventions, their geographical distribution, implementers, and funding schemes), we will provide descriptive statistics using frequency counts, percentages, and graphs (e.g., tree graphs or waffle charts) from the data extraction items. We will describe the number of peer-reviewed articles by country, region, or city, type of service, type of implementer, funding mechanism of the study and the service, and key population characteristics (such as primary substance use, and IDU practices, among others). We will later describe the studies, in terms of study design, sample size, year of data recollection, settings in which it was carried out, and primary results. We will include both intended and unintended outcomes reported in study findings, regardless of whether they have been quantitatively measured. We will describe all the services we identify in our records (e.g., housing, employment, etc.). We will then construct a heat map of the LAC region highlighting where HRS and interventions have been studied in LAC.

To respond to question number 5, we will conduct an inductive qualitative synthesis as proposed by Pollock et al. [41] to identify key policy, practice, and research gaps involving HRS and interventions in LAC. The information will be reviewed by two team members, who will develop a series of codes that allow summarizing the main emerging themes in the extracted articles (open coding phase). Then, the reviewers will meet to discuss the emerging themes and develop a coding framework, which will be discussed with the full research team. After approval of the coding framework, we will review each of the included documents and code accordingly. This will allow us to present a comprehensive synthesis of the services under study, as well as recurrent gaps in the region.

### Stage 6: Conducting consultations

We want to engage knowledge users [42], including additional experts and organizations of PWUD in LAC. To do this, we will send the results to organizations of PWUD in LAC, International Organizations, and three additional experts derived from the 10 most recent peer-reviewed articles that were included in our review. With this, we intend to seek their perspectives regarding the results and synthesis in terms of being relevant and meaningful [42]. With participants involved in these organizations, we will conduct one virtual focus group to discuss the findings and policy challenges for the different territories for the implementation of HRS and interventions for PWUD. A list of potential knowledge users is presented in the Appendix (S3 Table). The manuscript will be written in English, Spanish, and Portuguese.

### Pilot phase

To assess the feasibility of the scoping review, searches were conducted across all databases up to December 2024, yielding a total of 1,990 records, comprising 1,182 unique records. Later, IB and KB screened 381 records, of which 30 were included for full revision. The data sheet extraction was piloted and slightly modified after looking at three random studies that met the criteria. We estimate that data screening will be completed by October 2025, and data extraction in December 2025. Preliminary results are expected also for February 2026.

## Discussion

The proposed scoping review will provide valuable information regarding the current state of HRS and interventions for PWUD in LAC. To our knowledge, this is the first scoping review to map HRS and interventions in LAC using a systematic approach. Furthermore, the strengths of this review include the broad number of services, countries, and time assessed, as well as the consultation with experts and knowledge users to ensure its utility. This scoping review will also fill a knowledge gap in the current literature. For example, a review of literature on harm reduction strategies for the misuse of alcohol and other drugs was conducted by Gomes and Vecchia in 2018, but it focused on comparing services of the Brazilian context with those elsewhere [32]. Another review was conducted by Marín-Navarrete and colleagues [5] on the development and evaluation of treatment for SUDs in Latin America, but they only focused on treatment, included research published between July 2016 and December 2017 (18 months) and used now outdated information from the ATLAS on Substance Use (ATLAS-SU) project of the World Health Organization [43].

We acknowledge limitations to our scoping review. First, we will miss harm reduction efforts described in gray literature, which may provide rich data given the LAC context, where research is still in incipient stages. However, we will include six databases of scientific literature, including some based on the LAC region, as well as records in four languages. Based on the pilot screening phase, we believe our search will be comprehensive enough to provide an overview of both well-established and incipient research on HRS and interventions in the region. Second, our search strategy may have potentially missed other general community/health services that may contain some harm reduction components. Nevertheless, based on our pilot screening and extraction, we have found a wide variety of HRS and interventions, including some that are integrated into health systems and services that may not label themselves directly as harm reduction (e.g., street outreach clinics or Consultório na Rua in Brazil). Despite these limitations, the findings from this review can help guide future research for evaluating services that are already being implemented or unveiling services needed in the region. By focusing on services that are being already provided in LAC, we intend to disseminate the results of potential services into other low-resource settings in LAC. HRS and interventions are not just needed among PWID in LAC, who have been particularly affected by a lack of institutional responses to bloodborne diseases, like HIV [13], but also for those who use stimulants or alcohol, who make up the majority of people experiencing SUDs in the region. Moreover, the emergence of new synthetic and polysubstance-involving psychoactive substances poses important challenges for PWUD and healthcare providers in LAC, who historically have been exposed to harms associated with alcohol, cannabis, and cocaine. The analytical and forensic chemistry of substances is well-integrated with HR initiatives and clinical procedures in other regions [44,45], whereas in LAC this link is absent, worsening the situation. The changing and historical landscape of substance use in LAC needs further research and surveillance, which will allow a better design of new HRS and interventions for this region.

## Supporting information

S1 File Appendix. (DOCX)
