# Supplementary material for: Harm reduction services and interventions for People Who Use Drugs (PWUD) in Latin America and the Caribbean (LAC) between 2013–2024: A scoping review protocol
Source: PLoS One. 2025 Nov 24;20(11):e0334978. doi: 10.1371/journal.pone.0334978 (PMC12643314; doi:10.1371/journal.pone.0334978)
Supplement: S1 File — (DOCX) [file pone.0334978.s001.docx]

## Supporting information

**S1 Appendix.** PRISMA-P 2015 Checklist

This checklist has been adapted for use with protocol submissions to Systematic Reviews from Table 3 in Moher D et al: Preferred reporting items for systematic review and meta-analysis protocols (PRISMA-P) 2015 statement. Systematic Reviews 2015 4:1.

| **Section/topic** | **#** | **Checklist item** | **Information reported** | | **Line number(s)** |
| --- | --- | --- | --- | --- | --- |
|  |  |  | **Yes** | **No** |  |
| **ADMINISTRATIVE INFORMATION** | | | | | |
| **Title** | | | | | |
| Identification | 1a | Identify the report as a protocol of a systematic review |  |  | 3 |
| Update | 1b | If the protocol is for an update of a previous systematic review, identify as such |  |  | N/A |
| **Registration** | 2 | If registered, provide the name of the registry (e.g., PROSPERO) and registration number in the Abstract |  |  | 109 |
| **Authors** | | | | | |
| Contact | 3a | Provide name, institutional affiliation, and e-mail address of all protocol authors; provide physical mailing address of corresponding author |  |  | 5-16 |
| Contributions | 3b | Describe contributions of protocol authors and identify the guarantor of the review |  |  |  |
| **Amendments** | 4 | If the protocol represents an amendment of a previously completed or published protocol, identify as such and list changes; otherwise, state plan for documenting important protocol amendments |  |  | 110 |
| **Support** | | | | | |
| Sources | 5a | Indicate sources of financial or other support for the review |  |  | 24 |
| Sponsor | 5b | Provide name for the review funder and/or sponsor |  |  | 24 |
| Role of sponsor/funder | 5c | Describe roles of funder(s), sponsor(s), and/or institution(s), if any, in developing the protocol |  |  | 24 |
| **INTRODUCTION** | | | | | |
| **Rationale** | 6 | Describe the rationale for the review in the context of what is already known |  |  | 89-98 |
| **Objectives** | 7 | Provide an explicit statement of the question(s) the review will address with reference to participants, interventions, comparators, and outcomes (PICO) |  |  | 97-98;112-118 |
| **METHODS** | | | | | |
| **Eligibility criteria** | 8 | Specify the study characteristics (e.g., PICO, study design, setting, time frame) and report characteristics (e.g., years considered, language, publication status) to be used as criteria for eligibility for the review |  |  | 120-130 |
| **Information sources** | 9 | Describe all intended information sources (e.g., electronic databases, contact with study authors, trial registers, or other grey literature sources) with planned dates of coverage |  |  | 128-129;134-147 |
| **Search strategy** | 10 | Present draft of search strategy to be used for at least one electronic database, including planned limits, such that it could be repeated |  |  | 148 |
| ***STUDY RECORDS*** | | | | | |
| Data management | 11a | Describe the mechanism(s) that will be used to manage records and data throughout the review |  |  | 151-153 |
| Selection process | 11b | State the process that will be used for selecting studies (e.g., two independent reviewers) through each phase of the review (i.e., screening, eligibility, and inclusion in meta-analysis) |  |  | 152-164 |
| Data collection process | 11c | Describe planned method of extracting data from reports (e.g., piloting forms, done independently, in duplicate), any processes for obtaining and confirming data from investigators |  |  | 169-174 |
| **Data items** | 12 | List and define all variables for which data will be sought (e.g., PICO items, funding sources), any pre-planned data assumptions and simplifications |  |  | 180-190 |
| **Outcomes and prioritization** | 13 | List and define all outcomes for which data will be sought, including prioritization of main and additional outcomes, with rationale |  |  | N/A |
| **Risk of bias in individual studies** | 14 | Describe anticipated methods for assessing risk of bias of individual studies, including whether this will be done at the outcome or study level, or both; state how this information will be used in data synthesis |  |  | N/A |
| ***DATA*** | | | | | |
| **Synthesis** | 15a | Describe criteria under which study data will be quantitatively synthesized |  |  | 180-190 |
|  | 15b | If data are appropriate for quantitative synthesis, describe planned summary measures, methods of handling data, and methods of combining data from studies, including any planned exploration of consistency (e.g., *I* ^2^, Kendall’s tau) |  |  | N/A |
|  | 15c | Describe any proposed additional analyses (e.g., sensitivity or subgroup analyses, meta-regression) |  |  | N/A |
|  | 15d | If quantitative synthesis is not appropriate, describe the type of summary planned |  |  | 191-198 |
| **Meta-bias(es)** | 16 | Specify any planned assessment of meta-bias(es) (e.g., publication bias across studies, selective reporting within studies) |  |  | N/A |
| **Confidence in cumulative evidence** | 17 | Describe how the strength of the body of evidence will be assessed (e.g., GRADE) |  |  | N/A |

**S2 Appendix 2.** Countries and territories

**S1 Table.** List of countries and overseas territories

| Latin American Countries | Caribbean Countries | Overseas territories |
| --- | --- | --- |
| Argentina | Antigua and Barbuda | Anguilla |
| Belize | The Bahamas | Aruba |
| Bolivia | Barbados | Bermuda |
| Brazil | Cuba | British Virgin Islands |
| Chile | Dominica | Caribbean Netherlands |
| Colombia | Dominican Republic | Cayman Islands |
| Costa Rica | Grenada | Curaçao |
| Ecuador | Haiti | Falkland Islands |
| El Salvador | Jamaica | French Guiana |
| Guatemala | Saint Kitts and Nevis | Guadeloupe |
| Guyana | Saint Lucia | Martinique |
| Honduras | Saint Vincent and the Grenadines | Montserrat |
| Mexico | Trinidad and Tobago | Puerto Rico |
| Nicaragua |  | Saint Barthélemy |
| Panama |  | Saint Martin |
| Paraguay |  | South Georgia and the South Sandwich Islands |
| Peru |  | Turks and Caicos |
| Suriname |  | U.S. Virgin Islands |
| Uruguay |  |  |
| Venezuela |  |  |

**S3 Appendix. Databases, search terms and queries**

**S2 Table.** Keywords and queries

| **Database** | **Keywords and Queries** | **Number of records** |
| --- | --- | --- |
| **Pubmed** | (("Latin America"[tiab] OR "South America"[tiab] OR "Central America"[tiab] OR "Caribbean"[tiab] OR "Argentina"[tiab] OR "Belize"[tiab] OR "Bolivia"[tiab] OR "Brazil"[tiab] OR "Chile"[tiab] OR "Colombia"[tiab] OR "Costa Rica"[tiab] OR "Ecuador"[tiab] OR "El Salvador"[tiab] OR "Guatemala"[tiab] OR "Guyana"[tiab] OR "Honduras"[tiab] OR "Mexico"[tiab] OR "Nicaragua"[tiab] OR "Panama"[tiab] OR "Paraguay"[tiab] OR "Peru"[tiab] OR "Suriname"[tiab] OR "Uruguay"[tiab] OR "Venezuela"[tiab] OR "Antigua and Barbuda"[tiab] OR "The Bahamas"[tiab] OR "Barbados"[tiab] OR "Cuba"[tiab] OR "Dominica"[tiab] OR "Dominican Republic"[tiab] OR "Grenada"[tiab] OR "Haiti"[tiab] OR "Jamaica"[tiab] OR "Saint Kitts and Nevis"[tiab] OR "Saint Lucia"[tiab] OR "Saint Vincent and the Grenadines"[tiab] OR "Trinidad and Tobago"[tiab] OR "Anguilla"[tiab] OR "Aruba"[tiab] OR "Bermuda"[tiab] OR "British Virgin Islands"[tiab] OR "Caribbean Netherlands"[tiab] OR "Cayman Islands"[tiab] OR "Curaçao"[tiab] OR "Falkland Islands"[tiab] OR "French Guiana"[tiab] OR "Guadeloupe"[tiab] OR "Martinique"[tiab] OR "Montserrat"[tiab] OR "Puerto Rico"[tiab] OR "Saint Barthélemy"[tiab] OR "Saint Martin"[tiab] OR "South Georgia and the South Sandwich Islands"[tiab] OR "Turks and Caicos"[tiab] OR "U.S. Virgin Islands"[tiab] OR "Latin America"[Mesh] OR "Caribbean Region"[Mesh] OR "Central America"[Mesh] OR "South America"[Mesh])) AND (("People who use drug*"[tiab] OR "People who inject drug*"[tiab] OR "People who use drug*"[tiab] OR "PWID"[tiab] OR "PWUD"[tiab] OR "PWIDs"[tiab] OR "PWUDs"[tiab] OR "Substance Abuse"[tiab] OR "Substance Dependence"[tiab] OR "Substance Use Disorder*"[tiab] OR "Substance use"[tiab] OR "Alcohol*"[tiab] OR "Cannabi*"[tiab] OR "Marijuana*"[tiab] OR "Cocaine*"[tiab] OR "Freebase"[tiab] OR "Coca base"[tiab] OR "Coca"[tiab] OR "Coca paste"[tiab] OR "Crack cocaine"[tiab] OR "Amphetamine*"[tiab] OR "Methamphetamine*"[tiab] OR "MDMA"[tiab] OR "Ecstasy"[tiab] OR "Psychoactive substance*"[tiab] OR "Hallucinogen*"[tiab] OR "Ketamine*"[tiab] OR "Sedatives"[tiab] OR "Benzodiazepine*"[tiab] OR "Psilocybin*"[tiab] OR "LSD"[tiab] OR "Opioid*"[tiab] OR "AUD"[tiab] OR "OUD"[tiab] OR "SUD"[tiab] OR "Alcohol-Induced Disorders"[Mesh] OR "Alcohol-Related Disorders"[Mesh] OR "Amphetamine-Related Disorders"[Mesh] OR "Cocaine-Related Disorders"[Mesh] OR "Drug Users"[Mesh] OR "Inhalant Abuse"[Mesh] OR "Marijuana Abuse"[Mesh] OR "Opioid-Related Disorders"[Mesh] OR "Substance Withdrawal Syndrome"[Mesh] OR "Substance-Related Disorders"[Mesh])) AND (("Harm reduction"[tiab] OR "Risk reduction"[tiab] OR "Harm-reduction"[tiab] OR "Risk-reduction"[tiab] OR "Buprenorphine"[tiab] OR "Medication Assisted Treatment"[tiab] OR "Methadone"[tiab] OR "MMT"[tiab] OR "Naltrexone"[tiab] OR "Opiate Medication-Assisted Therap*"[tiab] OR "Opiate Medication-Assisted Treatment*"[tiab] OR "Opiate Replacement Therap*"[tiab] OR "Opiate Replacement Treatment*"[tiab] OR "Opiate Substitution Therap*"[tiab] OR "Opiate Substitution Treatment*"[tiab] OR "Opioid Medication-Assisted Therap*"[tiab] OR "Opioid Medication-Assisted Treatment*"[tiab] OR "Opioid Replacement Therap*"[tiab] OR "Opioid Replacement Treatment*"[tiab] OR "Opioid Substitution Therap*"[tiab] OR "Opioid Substitution Treatment*"[tiab] OR "OST"[tiab] OR "Needle and syringe program*"[tiab] OR "Needle Exchange*"[tiab] OR "Needle-Exchange*"[tiab] OR "NEP"[tiab] OR "SEP"[tiab] OR "SSP"[tiab] OR "Syringe Exchange*"[tiab] OR "Syringe Services*"[tiab] OR "Syringe-Exchange*"[tiab] OR "Drug consumption facilit*"[tiab] OR "Drug consumption room*"[tiab] OR "Drug consumption site*"[tiab] OR "Overdose prevention center*"[tiab] OR "Supervised consumption*"[tiab] OR "Supervised Injecting*"[tiab] OR "Drug Paraphernalia"[tiab] OR "Foil*"[tiab] OR "Mouthpiece*"[tiab] OR "Pipe*"[tiab] OR "Safer consumption equipment*"[tiab] OR "Safer consumption kit*"[tiab] OR "Safer consumption suppl*"[tiab] OR "Safer crack"[tiab] OR "Safer drug use"[tiab] OR "Safer smoking equipment*"[tiab] OR "Safer smoking kit*"[tiab] OR "Safer use equipment*"[tiab] OR "Safer use kit*"[tiab] OR "Safer use suppl*"[tiab] OR "Sterile consumption equipment*"[tiab] OR "Sterile consumption kit*"[tiab] OR "Sterile consumption suppl*"[tiab] OR "Sterile smoking equipment*"[tiab] OR "Sterile smoking kit*"[tiab] OR "Sterile use equipment*"[tiab] OR "Sterile use kit*"[tiab] OR "Sterile use suppl*"[tiab] OR "Alcohol management program*"[tiab] OR "Alcohol Moderation Management"[tiab] OR "Moderation Management"[tiab] OR "Managed alcohol program*"[tiab] OR "Managed alcohol"[tiab] OR "Alcohol Moderation"[tiab] OR "Community managed alcohol program"[tiab] OR "Drinking under control program*"[tiab] OR "Drinkers lounge"[tiab] OR "Managed alcohol administration"[tiab] OR "Drug checking*"[tiab] OR "Drug safety test*"[tiab] OR "Drug test*"[tiab] OR "Reagents*"[tiab] OR "Street drug analys*"[tiab] OR "Test strip*"[tiab] OR "Testing of illicit substance*"[tiab] OR "Testing of substance*"[tiab] OR "Harm Reduction"[Mesh] OR "Needle-Exchange Programs"[Mesh] OR "Opiate Substitution Treatment"[Mesh] OR "Community Health Services"[Mesh])) | 407 |
| **Web of Science** | ((((TI="Latin America" OR AB="Latin America") OR (TI="South America" OR AB="South America") OR (TI="Central America" OR AB="Central America") OR (TI=Caribbean OR AB=Caribbean) OR (TI=Argentina OR AB=Argentina) OR (TI=Belize OR AB=Belize) OR (TI=Bolivia OR AB=Bolivia) OR (TI=Brazil OR AB=Brazil) OR (TI=Chile OR AB=Chile) OR (TI=Colombia OR AB=Colombia) OR (TI="Costa Rica" OR AB="Costa Rica") OR (TI=Ecuador OR AB=Ecuador) OR (TI="El Salvador" OR AB="El Salvador") OR (TI=Guatemala OR AB=Guatemala) OR (TI=Guyana OR AB=Guyana) OR (TI=Honduras OR AB=Honduras) OR (TI=Mexico OR AB=Mexico) OR (TI=Nicaragua OR AB=Nicaragua) OR (TI=Panama OR AB=Panama) OR (TI=Paraguay OR AB=Paraguay) OR (TI=Peru OR AB=Peru) OR (TI=Suriname OR AB=Suriname) OR (TI=Uruguay OR AB=Uruguay) OR (TI=Venezuela OR AB=Venezuela) OR (TI="Antigua and Barbuda" OR AB="Antigua and Barbuda") OR (TI="The Bahamas" OR AB="The Bahamas") OR (TI=Barbados OR AB=Barbados) OR (TI=Cuba OR AB=Cuba) OR (TI=Dominica OR AB=Dominica) OR (TI="Dominican Republic" OR AB="Dominican Republic") OR (TI=Grenada OR AB=Grenada) OR (TI=Haiti OR AB=Haiti) OR (TI=Jamaica OR AB=Jamaica) OR (TI="Saint Kitts and Nevis" OR AB="Saint Kitts and Nevis") OR (TI="Saint Lucia" OR AB="Saint Lucia") OR (TI="Saint Vincent and the Grenadines" OR AB="Saint Vincent and the Grenadines") OR (TI="Trinidad and Tobago" OR AB="Trinidad and Tobago") OR (TI=Anguilla OR AB=Anguilla) OR (TI=Aruba OR AB=Aruba) OR (TI=Bermuda OR AB=Bermuda) OR (TI="British Virgin Islands" OR AB="British Virgin Islands") OR (TI="Caribbean Netherlands" OR AB="Caribbean Netherlands") OR (TI="Cayman Islands" OR AB="Cayman Islands") OR (TI=Curaçao OR AB=Curaçao) OR (TI="Falkland Islands" OR AB="Falkland Islands") OR (TI="French Guiana" OR AB="French Guiana") OR (TI=Guadeloupe OR AB=Guadeloupe) OR (TI=Martinique OR AB=Martinique) OR (TI=Montserrat OR AB=Montserrat) OR (TI="Puerto Rico" OR AB="Puerto Rico") OR (TI="Saint Barthélemy" OR AB="Saint Barthélemy") OR (TI="Saint Martin" OR AB="Saint Martin") OR (TI="South Georgia and the South Sandwich Islands" OR AB="South Georgia and the South Sandwich Islands") OR (TI="Turks and Caicos" OR AB="Turks and Caicos") OR (TI="U.S. Virgin Islands" OR AB="U.S. Virgin Islands") OR ALL="Latin America" OR ALL="Caribbean Region" OR ALL="Central America" OR ALL="South America")) AND (((TI="People who use drug*" OR AB="People who use drug*") OR (TI="People who inject drug*" OR AB="People who inject drug*") OR (TI="People who use drug*" OR AB="People who use drug*") OR (TI=PWID OR AB=PWID) OR (TI=PWUD OR AB=PWUD) OR (TI=PWIDs OR AB=PWIDs) OR (TI=PWUDs OR AB=PWUDs) OR (TI="Substance Abuse" OR AB="Substance Abuse") OR (TI="Substance Dependence" OR AB="Substance Dependence") OR (TI="Substance Use Disorder*" OR AB="Substance Use Disorder*") OR (TI="Substance use" OR AB="Substance use") OR (TI=Alcohol* OR AB=Alcohol*) OR (TI=Cannabi* OR AB=Cannabi*) OR (TI=Marijuana* OR AB=Marijuana*) OR (TI=Cocaine* OR AB=Cocaine*) OR (TI=Freebase OR AB=Freebase) OR (TI="Coca base" OR AB="Coca base") OR (TI=Coca OR AB=Coca) OR (TI="Coca paste" OR AB="Coca paste") OR (TI="Crack cocaine" OR AB="Crack cocaine") OR (TI=Amphetamine* OR AB=Amphetamine*) OR (TI=Methamphetamine* OR AB=Methamphetamine*) OR (TI=MDMA OR AB=MDMA) OR (TI=Ecstasy OR AB=Ecstasy) OR (TI="Psychoactive substance*" OR AB="Psychoactive substance*") OR (TI=Hallucinogen* OR AB=Hallucinogen*) OR (TI=Ketamine* OR AB=Ketamine*) OR (TI=Sedatives OR AB=Sedatives) OR (TI=Benzodiazepine* OR AB=Benzodiazepine*) OR (TI=Psilocybin* OR AB=Psilocybin*) OR (TI=LSD OR AB=LSD) OR (TI=Opioid* OR AB=Opioid*) OR (TI=AUD OR AB=AUD) OR (TI=OUD OR AB=OUD) OR (TI=SUD OR AB=SUD) OR ALL="Alcohol-Induced Disorders" OR ALL="Alcohol-Related Disorders" OR ALL="Amphetamine-Related Disorders" OR ALL="Cocaine-Related Disorders" OR ALL="Drug Users" OR ALL="Inhalant Abuse" OR ALL="Marijuana Abuse" OR ALL="Opioid-Related Disorders" OR ALL="Substance Withdrawal Syndrome" OR ALL="Substance-Related Disorders"))) AND (((TI="Harm reduction" OR AB="Harm reduction") OR (TI="Risk reduction" OR AB="Risk reduction") OR (TI=Harm-reduction OR AB=Harm-reduction) OR (TI=Risk-reduction OR AB=Risk-reduction) OR (TI=Buprenorphine OR AB=Buprenorphine) OR (TI="Medication Assisted Treatment" OR AB="Medication Assisted Treatment") OR (TI=Methadone OR AB=Methadone) OR (TI=MMT OR AB=MMT) OR (TI=Naltrexone OR AB=Naltrexone) OR (TI="Opiate Medication-Assisted Therap*" OR AB="Opiate Medication-Assisted Therap*") OR (TI="Opiate Medication-Assisted Treatment*" OR AB="Opiate Medication-Assisted Treatment*") OR (TI="Opiate Replacement Therap*" OR AB="Opiate Replacement Therap*") OR (TI="Opiate Replacement Treatment*" OR AB="Opiate Replacement Treatment*") OR (TI="Opiate Substitution Therap*" OR AB="Opiate Substitution Therap*") OR (TI="Opiate Substitution Treatment*" OR AB="Opiate Substitution Treatment*") OR (TI="Opioid Medication-Assisted Therap*" OR AB="Opioid Medication-Assisted Therap*") OR (TI="Opioid Medication-Assisted Treatment*" OR AB="Opioid Medication-Assisted Treatment*") OR (TI="Opioid Replacement Therap*" OR AB="Opioid Replacement Therap*") OR (TI="Opioid Replacement Treatment*" OR AB="Opioid Replacement Treatment*") OR (TI="Opioid Substitution Therap*" OR AB="Opioid Substitution Therap*") OR (TI="Opioid Substitution Treatment*" OR AB="Opioid Substitution Treatment*") OR (TI=OST OR AB=OST) OR (TI="Needle and syringe program*" OR AB="Needle and syringe program*") OR (TI="Needle Exchange*" OR AB="Needle Exchange*") OR (TI=Needle-Exchange* OR AB=Needle-Exchange*) OR (TI=NEP OR AB=NEP) OR (TI=SEP OR AB=SEP) OR (TI=SSP OR AB=SSP) OR (TI="Syringe Exchange*" OR AB="Syringe Exchange*") OR (TI="Syringe Services*" OR AB="Syringe Services*") OR (TI=Syringe-Exchange* OR AB=Syringe-Exchange*) OR (TI="Drug consumption facilit*" OR AB="Drug consumption facilit*") OR (TI="Drug consumption room*" OR AB="Drug consumption room*") OR (TI="Drug consumption site*" OR AB="Drug consumption site*") OR (TI="Overdose prevention center*" OR AB="Overdose prevention center*") OR (TI="Supervised consumption*" OR AB="Supervised consumption*") OR (TI="Supervised Injecting*" OR AB="Supervised Injecting*") OR (TI="Drug Paraphernalia" OR AB="Drug Paraphernalia") OR (TI=Foil* OR AB=Foil*) OR (TI=Mouthpiece* OR AB=Mouthpiece*) OR (TI=Pipe* OR AB=Pipe*) OR (TI="Safer consumption equipment*" OR AB="Safer consumption equipment*") OR (TI="Safer consumption kit*" OR AB="Safer consumption kit*") OR (TI="Safer consumption suppl*" OR AB="Safer consumption suppl*") OR (TI="Safer crack" OR AB="Safer crack") OR (TI="Safer drug use" OR AB="Safer drug use") OR (TI="Safer smoking equipment*" OR AB="Safer smoking equipment*") OR (TI="Safer smoking kit*" OR AB="Safer smoking kit*") OR (TI="Safer use equipment*" OR AB="Safer use equipment*") OR (TI="Safer use kit*" OR AB="Safer use kit*") OR (TI="Safer use suppl*" OR AB="Safer use suppl*") OR (TI="Sterile consumption equipment*" OR AB="Sterile consumption equipment*") OR (TI="Sterile consumption kit*" OR AB="Sterile consumption kit*") OR (TI="Sterile consumption suppl*" OR AB="Sterile consumption suppl*") OR (TI="Sterile smoking equipment*" OR AB="Sterile smoking equipment*") OR (TI="Sterile smoking kit*" OR AB="Sterile smoking kit*") OR (TI="Sterile use equipment*" OR AB="Sterile use equipment*") OR (TI="Sterile use kit*" OR AB="Sterile use kit*") OR (TI="Sterile use suppl*" OR AB="Sterile use suppl*") OR (TI="Alcohol management program*" OR AB="Alcohol management program*") OR (TI="Alcohol Moderation Management" OR AB="Alcohol Moderation Management") OR (TI="Moderation Management" OR AB="Moderation Management") OR (TI="Managed alcohol program*" OR AB="Managed alcohol program*") OR (TI="Managed alcohol" OR AB="Managed alcohol") OR (TI="Alcohol Moderation" OR AB="Alcohol Moderation") OR (TI="Community managed alcohol program" OR AB="Community managed alcohol program") OR (TI="Drinking under control program*" OR AB="Drinking under control program*") OR (TI="Drinkers lounge" OR AB="Drinkers lounge") OR (TI="Managed alcohol administration" OR AB="Managed alcohol administration") OR (TI="Drug checking*" OR AB="Drug checking*") OR (TI="Drug safety test*" OR AB="Drug safety test*") OR (TI="Drug test*" OR AB="Drug test*") OR (TI=Reagents* OR AB=Reagents*) OR (TI="Street drug analys*" OR AB="Street drug analys*") OR (TI="Test strip*" OR AB="Test strip*") OR (TI="Testing of illicit substance*" OR AB="Testing of illicit substance*") OR (TI="Testing of substance*" OR AB="Testing of substance*") OR ALL="Harm Reduction" OR ALL="Needle-Exchange Programs" OR ALL="Opiate Substitution Treatment" OR ALL="Community Health Services")) | 358 |
| **Scopus** | (((TITLE-ABS("Latin America") OR TITLE-ABS("South America") OR TITLE-ABS("Central America") OR TITLE-ABS(Caribbean) OR TITLE-ABS(Argentina) OR TITLE-ABS(Belize) OR TITLE-ABS(Bolivia) OR TITLE-ABS(Brazil) OR TITLE-ABS(Chile) OR TITLE-ABS(Colombia) OR TITLE-ABS("Costa Rica") OR TITLE-ABS(Ecuador) OR TITLE-ABS("El Salvador") OR TITLE-ABS(Guatemala) OR TITLE-ABS(Guyana) OR TITLE-ABS(Honduras) OR TITLE-ABS(Mexico) OR TITLE-ABS(Nicaragua) OR TITLE-ABS(Panama) OR TITLE-ABS(Paraguay) OR TITLE-ABS(Peru) OR TITLE-ABS(Suriname) OR TITLE-ABS(Uruguay) OR TITLE-ABS(Venezuela) OR TITLE-ABS("Antigua and Barbuda") OR TITLE-ABS("The Bahamas") OR TITLE-ABS(Barbados) OR TITLE-ABS(Cuba) OR TITLE-ABS(Dominica) OR TITLE-ABS("Dominican Republic") OR TITLE-ABS(Grenada) OR TITLE-ABS(Haiti) OR TITLE-ABS(Jamaica) OR TITLE-ABS("Saint Kitts and Nevis") OR TITLE-ABS("Saint Lucia") OR TITLE-ABS("Saint Vincent and the Grenadines") OR TITLE-ABS("Trinidad and Tobago") OR TITLE-ABS(Anguilla) OR TITLE-ABS(Aruba) OR TITLE-ABS(Bermuda) OR TITLE-ABS("British Virgin Islands") OR TITLE-ABS("Caribbean Netherlands") OR TITLE-ABS("Cayman Islands") OR TITLE-ABS(Curaçao) OR TITLE-ABS("Falkland Islands") OR TITLE-ABS("French Guiana") OR TITLE-ABS(Guadeloupe) OR TITLE-ABS(Martinique) OR TITLE-ABS(Montserrat) OR TITLE-ABS("Puerto Rico") OR TITLE-ABS("Saint Barthélemy") OR TITLE-ABS("Saint Martin") OR TITLE-ABS("South Georgia and the South Sandwich Islands") OR TITLE-ABS("Turks and Caicos") OR TITLE-ABS("U.S. Virgin Islands") OR INDEXTERMS("Latin America") OR INDEXTERMS("Caribbean Region") OR INDEXTERMS("Central America") OR INDEXTERMS("South America"))) AND ((TITLE-ABS("People who use drug*") OR TITLE-ABS("People who inject drug*") OR TITLE-ABS("People who use drug*") OR TITLE-ABS(PWID) OR TITLE-ABS(PWUD) OR TITLE-ABS(PWIDs) OR TITLE-ABS(PWUDs) OR TITLE-ABS("Substance Abuse") OR TITLE-ABS("Substance Dependence") OR TITLE-ABS("Substance Use Disorder*") OR TITLE-ABS("Substance use") OR TITLE-ABS(Alcohol*) OR TITLE-ABS(Cannabi*) OR TITLE-ABS(Marijuana*) OR TITLE-ABS(Cocaine*) OR TITLE-ABS(Freebase) OR TITLE-ABS("Coca base") OR TITLE-ABS(Coca) OR TITLE-ABS("Coca paste") OR TITLE-ABS("Crack cocaine") OR TITLE-ABS(Amphetamine*) OR TITLE-ABS(Methamphetamine*) OR TITLE-ABS(MDMA) OR TITLE-ABS(Ecstasy) OR TITLE-ABS("Psychoactive substance*") OR TITLE-ABS(Hallucinogen*) OR TITLE-ABS(Ketamine*) OR TITLE-ABS(Sedatives) OR TITLE-ABS(Benzodiazepine*) OR TITLE-ABS(Psilocybin*) OR TITLE-ABS(LSD) OR TITLE-ABS(Opioid*) OR TITLE-ABS(AUD) OR TITLE-ABS(OUD) OR TITLE-ABS(SUD) OR INDEXTERMS("Alcohol-Induced Disorders") OR INDEXTERMS("Alcohol-Related Disorders") OR INDEXTERMS("Amphetamine-Related Disorders") OR INDEXTERMS("Cocaine-Related Disorders") OR INDEXTERMS("Drug Users") OR INDEXTERMS("Inhalant Abuse") OR INDEXTERMS("Marijuana Abuse") OR INDEXTERMS("Opioid-Related Disorders") OR INDEXTERMS("Substance Withdrawal Syndrome") OR INDEXTERMS("Substance-Related Disorders")))) AND ((TITLE-ABS("Harm reduction") OR TITLE-ABS("Risk reduction") OR TITLE-ABS(Harm-reduction) OR TITLE-ABS(Risk-reduction) OR TITLE-ABS(Buprenorphine) OR TITLE-ABS("Medication Assisted Treatment") OR TITLE-ABS(Methadone) OR TITLE-ABS(MMT) OR TITLE-ABS(Naltrexone) OR TITLE-ABS("Opiate Medication-Assisted Therap*") OR TITLE-ABS("Opiate Medication-Assisted Treatment*") OR TITLE-ABS("Opiate Replacement Therap*") OR TITLE-ABS("Opiate Replacement Treatment*") OR TITLE-ABS("Opiate Substitution Therap*") OR TITLE-ABS("Opiate Substitution Treatment*") OR TITLE-ABS("Opioid Medication-Assisted Therap*") OR TITLE-ABS("Opioid Medication-Assisted Treatment*") OR TITLE-ABS("Opioid Replacement Therap*") OR TITLE-ABS("Opioid Replacement Treatment*") OR TITLE-ABS("Opioid Substitution Therap*") OR TITLE-ABS("Opioid Substitution Treatment*") OR TITLE-ABS(OST) OR TITLE-ABS("Needle and syringe program*") OR TITLE-ABS("Needle Exchange*") OR TITLE-ABS(Needle-Exchange*) OR TITLE-ABS(NEP) OR TITLE-ABS(SEP) OR TITLE-ABS(SSP) OR TITLE-ABS("Syringe Exchange*") OR TITLE-ABS("Syringe Services*") OR TITLE-ABS(Syringe-Exchange*) OR TITLE-ABS("Drug consumption facilit*") OR TITLE-ABS("Drug consumption room*") OR TITLE-ABS("Drug consumption site*") OR TITLE-ABS("Overdose prevention center*") OR TITLE-ABS("Supervised consumption*") OR TITLE-ABS("Supervised Injecting*") OR TITLE-ABS("Drug Paraphernalia") OR TITLE-ABS(Foil*) OR TITLE-ABS(Mouthpiece*) OR TITLE-ABS(Pipe*) OR TITLE-ABS("Safer consumption equipment*") OR TITLE-ABS("Safer consumption kit*") OR TITLE-ABS("Safer consumption suppl*") OR TITLE-ABS("Safer crack") OR TITLE-ABS("Safer drug use") OR TITLE-ABS("Safer smoking equipment*") OR TITLE-ABS("Safer smoking kit*") OR TITLE-ABS("Safer use equipment*") OR TITLE-ABS("Safer use kit*") OR TITLE-ABS("Safer use suppl*") OR TITLE-ABS("Sterile consumption equipment*") OR TITLE-ABS("Sterile consumption kit*") OR TITLE-ABS("Sterile consumption suppl*") OR TITLE-ABS("Sterile smoking equipment*") OR TITLE-ABS("Sterile smoking kit*") OR TITLE-ABS("Sterile use equipment*") OR TITLE-ABS("Sterile use kit*") OR TITLE-ABS("Sterile use suppl*") OR TITLE-ABS("Alcohol management program*") OR TITLE-ABS("Alcohol Moderation Management") OR TITLE-ABS("Moderation Management") OR TITLE-ABS("Managed alcohol program*") OR TITLE-ABS("Managed alcohol") OR TITLE-ABS("Alcohol Moderation") OR TITLE-ABS("Community managed alcohol program") OR TITLE-ABS("Drinking under control program*") OR TITLE-ABS("Drinkers lounge") OR TITLE-ABS("Managed alcohol administration") OR TITLE-ABS("Drug checking*") OR TITLE-ABS("Drug safety test*") OR TITLE-ABS("Drug test*") OR TITLE-ABS(Reagents*) OR TITLE-ABS("Street drug analys*") OR TITLE-ABS("Test strip*") OR TITLE-ABS("Testing of illicit substance*") OR TITLE-ABS("Testing of substance*") OR INDEXTERMS("Harm Reduction") OR INDEXTERMS("Needle-Exchange Programs") OR INDEXTERMS("Opiate Substitution Treatment") OR INDEXTERMS("Community Health Services"))) | 394 |
| **SciELO Spanish** | ((América Latina) OR (América del Sur) OR (América Central) OR (Caribe) OR (Argentina) OR (Antigua y Barbuda) OR (Anguila) OR (Belice) OR (Las Bahamas) OR (Aruba) OR (Bolivia) OR (Barbados) OR (Bermudas) OR (Brasil) OR (Cuba) OR (Islas Vírgenes Británicas) OR (Chile) OR (Dominica) OR (Países Bajos Caribeños) OR (Colombia) OR (República Dominicana) OR (Islas Caimán) OR (Costa Rica) OR (Granada) OR (Curazao) OR (Ecuador) OR (Haití) OR (Islas Malvinas) OR (El Salvador) OR (Jamaica) OR (Guayana Francesa) OR (Guatemala) OR (San Cristóbal y Nieves) OR (Guadalupe) OR (Guyana) OR (Santa Lucía) OR (Martinica) OR (Honduras) OR (San Vicente y las Granadinas) OR (Montserrat) OR (México) OR (Trinidad y Tobago) OR (Puerto Rico) OR (Nicaragua) OR (San Bartolomé) OR (Panamá) OR (San Martín) OR (Paraguay) OR (Islas Georgias del Sur y Sandwich del Sur) OR (Perú) OR (Islas Turcas y Caicos) OR (Surinam) OR (Islas Vírgenes de EE. UU.) OR (Uruguay) OR (Venezuela)) AND ((Trastornos relacionados con sustancias) OR (Alcohol*) OR (Marijuana*) OR (Marihuana*) OR (Pasta base) OR (Basoco) OR (Bazuco) OR (Coca base) OR (Cocaína*) OR (Crack) OR (Amfetamina*) OR (Metamfetamina*) OR (MDMA*) OR (Éxtasis*) OR (Sustancias psicoactiva*) OR (Alucinógen*) OR (Ketamina*) OR (Sedativos*) OR (Benzodiacepina*) OR (Psilocybin*) OR (LSD*) OR (Opiod*) OR (Opiáceo*)) AND ((Reducción del daño*) OR (Reducción de riesgo*) OR (Buprenorfina) OR (Tratamiento de sustitución de opiáceo*) OR (Metadona) OR (Naltrexona) OR (Intercambio de aguja*) OR (Intercambio de jeringa*) OR (Sala de consumo supervisado) OR (Centro de prevención de sobredosis) OR (Consumo seguro) OR (Uso de drogas seguro) OR (Consumo estéril) OR (Programa de manejo de alcohol*) OR (Programa de moderación) OR (Manejo de alcohol*) OR (Moderación de alcohol*) OR (Detección de sustancia*) OR (Análisis de sustancia*) OR (Servicio de análisis de sustancia*) OR (Testeo de sustancia*)) | 212 |
| **BIREME Spanish** | (("América Latina" OR "América del Sur" OR "América Central" OR "Caribe" OR "Argentina" OR "Antigua y Barbuda" OR "Anguila" OR "Belice" OR "Las Bahamas" OR "Aruba" OR "Bolivia" OR "Barbados" OR "Bermudas" OR "Brasil" OR "Cuba" OR "Islas Vírgenes Británicas" OR "Chile" OR "Dominica" OR "Países Bajos Caribeños" OR "Colombia" OR "República Dominicana" OR "Islas Caimán" OR "Costa Rica" OR "Granada" OR "Curazao" OR "Ecuador" OR "Haití" OR "Islas Malvinas" OR "El Salvador" OR "Jamaica" OR "Guayana Francesa" OR "Guatemala" OR "San Cristóbal y Nieves" OR "Guadalupe" OR "Guyana" OR "Santa Lucía" OR "Martinica" OR "Honduras" OR "San Vicente y las Granadinas" OR "Montserrat" OR "México" OR "Trinidad y Tobago" OR "Puerto Rico" OR "Nicaragua" OR "San Bartolomé" OR "Panamá" OR "San Martín" OR "Paraguay" OR "Islas Georgias del Sur y Sandwich del Sur" OR "Perú" OR "Islas Turcas y Caicos" OR "Surinam" OR "Islas Vírgenes de EE. UU." OR "Uruguay" OR "Venezuela") AND ("Trastornos relacionados con sustancias" OR "Alcohol*" OR "Marijuana*" OR "Marihuana*" OR "Pasta base" OR "Basoco" OR "Bazuco" OR "Coca base" OR "Cocaína*" OR "Crack" OR "Amfetamina*" OR "Metamfetamina*" OR "MDMA*" OR "Éxtasis*" OR "Sustancias psicoactiva*" OR "Alucinógen*" OR "Ketamina*" OR "Sedativos*" OR "Benzodiacepina*" OR "Psilocybin*" OR "LSD*" OR "Opioid*" OR "Opiáceo*") AND ("Reducción del daño*" OR "Reducción de riesgo*" OR "Buprenorfina" OR "Tratamiento de sustitución de opiáceo*" OR "Metadona" OR "Naltrexona" OR "Intercambio de aguja*" OR "Intercambio de jeringa*" OR "Sala de consumo supervisado" OR "Centro de prevención de sobredosis" OR "Consumo seguro" OR "Uso de drogas seguro" OR "Consumo estéril" OR "Programa de manejo de alcohol*" OR "Programa de moderación" OR "Manejo de alcohol*" OR "Moderación de alcohol*" OR "Detección de sustancia*" OR "Análisis de sustancia*" OR "Servicio de análisis de sustancia*" OR "Reagente*" OR "Testeo de sustancia*")) | 265 |
| **SciELO Portuguese** | ((América Latina) OR (América do Sul) OR (América Central) OR (Caribe) OR (Argentina) OR (Antígua e Barbuda) OR (Anguila) OR (Belize) OR (As Bahamas) OR (Aruba) OR (Bolívia) OR (Barbados) OR (Bermudas) OR (Brasil) OR (Cuba) OR (Ilhas Virgens Britânicas) OR (Chile) OR (Dominica) OR (Países Baixos Caribenhos) OR (Colômbia) OR (República Dominicana) OR (Ilhas Cayman) OR (Costa Rica) OR (Granada) OR (Curaçao) OR (Equador) OR (Haiti) OR (Ilhas Malvinas) OR (El Salvador) OR (Jamaica) OR (Guiana Francesa) OR (Guatemala) OR (São Cristóvão e Neves) OR (Guadalupe) OR (Guiana) OR (Santa Lúcia) OR (Martinica) OR (Honduras) OR (São Vicente e Granadinas) OR (Montserrat) OR (México) OR (Trinidad e Tobago) OR (Porto Rico) OR (Nicarágua) OR (São Bartolomeu) OR (Panamá) OR (São Martinho) OR (Paraguai) OR (Ilhas Geórgia do Sul e Sandwich do Sul) OR (Peru) OR (Ilhas Turcas e Caicos) OR (Suriname) OR (Ilhas Virgens dos EUA) OR (Uruguai) OR (Venezuela)) AND ((Transtornos Relacionados ao Uso de*) OR (Álcool*) OR (Marijuana*) OR (Maconha*) OR (Pasta base) OR (Basoco) OR (Bazuco) OR (Coca base) OR (Cocaína*) OR (Crack) OR (Anfetamina*) OR (Metanfetamina*) OR (MDMA*) OR (Êxtase*) OR (Substâncias psicoativas*) OR (Alucinógen*) OR (Cetamina*) OR (Sedativos*) OR (Benzodiazepina*) OR (Psilocibina*) OR (LSD*) OR (Opioide*) OR (Opiáceo*)) AND ((Redução de dano*) OR (Comportamento de Redução do Risco*) OR (Buprenorfina) OR (Tratamento de substituição de opiáceo*) OR (Metadona) OR (Naltrexona) OR (Programas de Troca de Agulhas) OR (Sala de consumo supervisionado) OR (Instalações de consumo supervisionado de drogas) OR (Centro de prevenção de overdose) OR (Consumo seguro) OR (Uso seguro de drogas) OR (Consumo estéril) OR (Programa de gerenciamento de álcool*) OR (Programa de moderação) OR (Gerenciamento de álcool*) OR (Moderação de álcool*) OR (Detecção de substância*) OR (Análise de substância*) OR (Serviço de análise de substância*) OR (Reagente*) OR (Detecção do Abuso de Substâncias*)) | 265 |
| **BIREME Portuguese** | (("América Latina" OR "América do Sul" OR "América Central" OR "Caribe" OR "Argentina" OR "Antígua e Barbuda" OR "Anguila" OR "Belize" OR "As Bahamas" OR "Aruba" OR "Bolívia" OR "Barbados" OR "Bermudas" OR "Brasil" OR "Cuba" OR "Ilhas Virgens Britânicas" OR "Chile" OR "Dominica" OR "Países Baixos Caribenhos" OR "Colômbia" OR "República Dominicana" OR "Ilhas Cayman" OR "Costa Rica" OR "Granada" OR "Curaçao" OR "Equador" OR "Haiti" OR "Ilhas Malvinas" OR "El Salvador" OR "Jamaica" OR "Guiana Francesa" OR "Guatemala" OR "São Cristóvão e Neves" OR "Guadalupe" OR "Guiana" OR "Santa Lúcia" OR "Martinica" OR "Honduras" OR "São Vicente e Granadinas" OR "Montserrat" OR "México" OR "Trinidad e Tobago" OR "Porto Rico" OR "Nicarágua" OR "São Bartolomeu" OR "Panamá" OR "São Martinho" OR "Paraguai" OR "Ilhas Geórgia do Sul e Sandwich do Sul" OR "Peru" OR "Ilhas Turcas e Caicos" OR "Suriname" OR "Ilhas Virgens dos EUA" OR "Uruguai" OR "Venezuela") AND (" Transtornos Relacionados ao Uso de*" OR "Álcool*" OR "Marijuana*" OR "Maconha*" OR "Pasta base" OR "Basoco" OR "Bazuco" OR "Coca base" OR "Cocaína*" OR "Crack" OR "Anfetamina*" OR "Metanfetamina*" OR "MDMA*" OR "Êxtase*" OR "Substâncias psicoativas*" OR "Alucinógen*" OR "Cetamina*" OR "Sedativos*" OR "Benzodiazepina*" OR "Psilocibina*" OR "LSD*" OR "Opioide*" OR "Opiáceo*") AND ("Redução de dano*" OR " Comportamento de Redução do Risco*" OR "Buprenorfina" OR "Tratamento de substituição de opiáceo*" OR "Metadona" OR "Naltrexona" OR "Programas de Troca de Agulhas*" OR "Sala de consumo supervisionado" OR “Instalações de consumo supervisionado de drogas” OR "Centro de prevenção de overdose" OR "Consumo seguro" OR "Uso seguro de drogas" OR "Consumo estéril" OR "Programa de gerenciamento de álcool*" OR "Programa de moderação" OR "Gerenciamento de álcool*" OR "Moderação de álcool*" OR "Detecção de substância*" OR "Análise de substância*" OR "Serviço de análise de substância*" OR "Reagente*" OR " Detecção do Abuso de Substâncias*")) | 11 |
| **French BIREME** | (("Amérique latine" OR "Amérique du Sud" OR "Amérique centrale" OR "Caraïbes" OR "Argentine" OR "Antigua-et-Barbuda" OR "Anguilla" OR "Belize" OR "Les Bahamas" OR "Aruba" OR "Bolivie" OR "Barbade" OR "Bermudes" OR "Brésil" OR "Cuba" OR "Îles Vierges britanniques" OR "Chili" OR "Dominique" OR "Pays-Bas caribéens" OR "Colombie" OR "République dominicaine" OR "Îles Caïmans" OR "Costa Rica" OR "Grenade" OR "Curaçao" OR "Équateur" OR "Haïti" OR "Îles Malouines" OR "Salvador" OR "Jamaïque" OR "Guyane française" OR "Guatemala" OR "Saint-Christophe-et-Niévès" OR "Guadeloupe" OR "Guyana" OR "Sainte-Lucie" OR "Martinique" OR "Honduras" OR "Saint-Vincent-et-les-Grenadines" OR "Montserrat" OR "Mexique" OR "Trinité-et-Tobago" OR "Porto Rico" OR "Nicaragua" OR "Saint-Barthélemy" OR "Panama" OR "Saint-Martin" OR "Paraguay" OR "Îles Géorgie du Sud et Sandwich du Sud" OR "Pérou" OR "Îles Turques-et-Caïques" OR "Suriname" OR "Îles Vierges des États-Unis" OR "Uruguay" OR "Venezuela") AND ("Troubles liés à une substance" OR "Alcool*" OR "Marijuana*" OR "Marihuana*" OR "Pâte base" OR "Basoco" OR "Bazuco" OR "Coca base" OR "Cocaïne*" OR "Crack" OR "Amphétamine*" OR "Méthamphétamine*" OR "MDMA*" OR "Ecstasy*" OR "Substances psychoactives*" OR "Hallucinogène*" OR "Kétamine*" OR "Sédatifs*" OR "Benzodiazépine*" OR "Psilocybine*" OR "LSD*" OR "Opioïde*" OR "Opiacé*") AND ("Réduction des dommages*" OR "Réduction des risques*" OR "Buprénorphine" OR "Traitement de substitution aux opiacés*" OR "Méthadone" OR "Naltrexone" OR "Partage de seringue*" OR "Échange d’aiguilles*" OR "Sites de consommation supervisée*" OR "Salles de consommation de drogue*" OR "Consommation sécurisée" OR "Utilisation sécurisée des drogues" OR "Consommation stérile" OR "Programme de gestion de l’alcool*" OR "Programme de modération" OR "Gestion de l’alcool*" OR "Modération de l’alcool*" OR "Détection de substances*" OR "Analyse de substances*" OR "Service de vérifications de substances*" OR "Test de substances*" OR "Service d'analyse des drogues*")) | 77 |

**S4 Appendix.**

**S3 Table.** List of organizations and locations

| Organization Name | Country(ies) |
| --- | --- |
| Latin American Network of People who Use Drugs (LANPUD) | Multiple |
| American States Organization (OEA) | Multiple |
| Instituto Iberoamericano de Reducción de Daños | Multiple |
| Harm Reduction International | Multiple |
| Ibero-American Network of NGOs working on Drugs and Other Addictions (RIOD) | Multiple |
| Asociación de reducción de daños de Argentina (ARDA) | Argentina |
| Red Argentina para los derechos y asistencia de las personas que usan Drogas (RADAUD) | Argentina |
| Red nacional de feministas antiprohibicionistas (RENFA) | Brazil |
| Centro É de Lei | Brazil |
| Rede Brasileira de Redução de Danos | Brazil |
| Escola Livre de Redução de Danos | Brazil |
| Movimentos_ | Brazil |
| Governo do Estado da Bahia – Programa Corra para o Abraço | Brazil |
| Reduciendo Daño | Chile |
| Acción Técnica Social | Colombia |
| Échele Cabeza | Colombia |
| ReverdeSer | México |
| Espolea | México |
| Latinoamérica por una Política Sensata de Drogas | México |
| Integracion Social Verter A.C. | México |
| Proyecto SOMA | Perú |
| Intercambios Puerto Rico | Puerto Rico |
